# Heat Release by Isolated Mouse Brain Mitochondria Detected with Diamond Thermometer

**DOI:** 10.3390/nano13010098

**Published:** 2022-12-25

**Authors:** Alexey M. Romshin, Alexander A. Osypov, Irina Yu. Popova, Vadim E. Zeeb, Andrey G. Sinogeykin, Igor I. Vlasov

**Affiliations:** 1 Prokhorov General Physics Institute of the Russian Academy of Sciences, 119991 Moscow, Russia; 2 Institute of Theoretical and Experimental Biophysics of the Russian Academy of Sciences, 142292 Moscow, Russia; 3 Institute of Higher Nervous Activity and Neurophysiology of the Russian Academy of Sciences, 117485 Moscow, Russia; 4 Wonder Technologies LLC, 143026 Moscow, Russia

**Keywords:** diamond thermometer, mitochondria, cell thermodynamics

## Abstract

The production of heat by mitochondria is critical for maintaining body temperature, regulating metabolic rate, and preventing oxidative damage to mitochondria and cells. Until the present, mitochondrial heat production has been characterized only by methods based on fluorescent probes, which are sensitive to environmental variations (viscosity, pH, ionic strength, quenching, etc.). Here, for the first time, the heat release of isolated mitochondria was unambiguously measured by a diamond thermometer (DT), which is absolutely indifferent to external non-thermal parameters. We show that during total uncoupling of transmembrane potential by CCCP application, the temperature near the mitochondria rises by 4–22 °C above the ambient temperature with an absolute maximum of 45 °C. Such a broad variation in the temperature response is associated with the heterogeneity of the mitochondria themselves as well as their aggregations in the isolated suspension. Spontaneous temperature bursts with comparable amplitude were also detected prior to CCCP application, which may reflect involvement of some mitochondria to ATP synthesis or membrane potential leaking to avoid hyperproduction of reactive oxygen species. The results obtained with the diamond temperature sensor shed light on the “hot mitochondria” paradox.

## 1. Introduction

Experimental detection of heat production in a single living cell attributed to its different compartments, organelles, ionic pumps, and ionic channels has long been a cutting edge challenge for modern experimental approaches in cell physiology research. The proven existence of sharp steady-state temperature gradients (tens of degrees °C) in submicrometer watery volumes near a nanoscale heat source [1] puts forward a beneficial base for the thermal signaling concept [2], supposing that living cells are capable of creating significant temperature gradients at nanoscale, engaging these ultralocal thermodynamic events as a variable driving force to govern cascades of morphological, biochemical, and electrical intracellular processes. Therefore, experimental uncovering of the signaling role of the nanoscale thermodynamic events bound to biochemical processes and the thermodynamics around ionic channels and pumps has become an ultimate priority. Being the ubiquitous and specialized factory for transforming the most powerful chemical energy supply of the aerobic respiration process to the universal cellular energy source in the form of macroergic phosphate bonds of ATP, mitochondria are natural producers of heat that is generated due to inefficient (up to 60 percent) energy conversion [3]. Together with a decoupling mechanism that is used to control the upper value of the proton potential to avoid excessive production of reactive oxygen species [4], this served as a preadaptation to the development of homeothermy, which maintains the organism’s homeostasis and intensifies metabolism at an unprecedented level. This is taken to the extreme in the brown fat of neonatants and hibernating animals, where mitochondria seem to be solely dedicated to heat production [5].

Various cellular dysfunctions and pathological conditions lead to changes in the energy metabolism of mitochondria [6] and, as a result, to changes in their heat production [7]. There have already been studies on the medical use of the ability of mitochondria to create heat. Recently, thermosensitive nanocarriers have been developed for targeted drug delivery to mitochondria [8]. These nanocarriers are able to enhance the accumulation of anticancer drugs in mitochondria at a certain endogenous mitochondrial temperature. This increases the selectivity for cancer cells and helps to reverse cancer drug resistance [9]. Despite these facts, there are still unresolved questions about the absolute temperature of mitochondria in different cells/tissues/organs/organisms, the range of these temperature fluctuations, and how these fluctuations depend on the functional state in normal and pathological conditions.

A comprehensive understanding of mitochondria thermogenesis requires a reliable and robust technique without an external biological impact. Recently, a great variety of techniques and approaches aimed at detecting mitochondrial temperature including fluorescent nanogels [9,10], polymers [11], and proteins [12,13,14]; molecular dyes [15,16,17,18]; and colloidal quantum dots [19] were elaborated. A number of them using probes located outside of mitochondria (extramitochondrial) reported increases in temperature of several degrees caused by enhanced mitochondrial activity, generally, due to uncoupling of the respiratory chain which boosts mitochondrial thermogenesis. Some other approaches based on thermosensors located within mitochondria (intramitochondrial) were able to identify an increase in the organelle temperature of several degrees in the same (or similar) conditions, suggesting that the mitochondrial radiators could be warmer than their surroundings. Although Chr’etien et al. [15] revealed a mitochondrial temperature of more than 10 °C above that of the culture medium in normal conditions (without any stimuli), their study remains so far the only evidence of such a high heat release by mitochondria.

Despite numerous experimental works supporting the hypothesis of “hot mitochondria” [20,21], the physical and biological mechanisms for furnishing perceptible thermal bursts remain a matter of controversy. So, Baffou et al. argued that according to Fourier’s law, hardly such a small organelle (typically 500 nm in diameter) could heat the intracellular surrounding by more than 0.1 mK [21]. Filling the temperature gap would necessitate organelles to produce the heat power P=1μW. Apparently, the mitochondria do not have enough fuel to meet this requirement. Baffou et. al. claimed that the existing fluorescent thermosensors do not directly measure the temperature, and, as with any fluorescent probe, are prone to many artifacts caused by variations in environment (viscosity, pH, ionic strength, quenching). Therefore, the validity of presented results along with their careful interpretation should be questioned.

In this paper, we employ a diamond thermometer (DT) designed by us for unambiguous extramitochondrial temperature measurements of isolated mouse brain mitochondria. The DT based on a single fluorescent diamond microparticle fixed at the tip of the glass capillary and pre-calibrated by temperature is absolutely insensitive to external non-thermal parameters allowing for unambiguous temperature detection. By means of DT instrumentation we demonstrate that the mitochondria isolated from the mouse brain could be several tens of degrees warmer than the surrounding environment at room temperature during natural metabolic processes as well as under membrane permeability agent application. The simple physical model is also provided to support the obtained results.

## 2. Materials and Methods

All animal protocols and experimental procedures were performed in accordance with the requirements of Directive 2010/63/EC of the European Union and Order of the Ministry of Health of Russia of 19 June 2003 No. 267 and were approved by the Ethics Committees for Animal Experimentation at the ITEB RAS. BALB/c mice (25–33 g) were purchased from the Stolbovaya vivarium of laboratory animals (https://www.pitst.ru (accessed on 1 January 2020), Moscow region, Russia) and housed with food and water ad libitum.

### 2.1. Mouse Brain Mitochondria Isolation

Adult mice (*n* = 6) were deeply anesthetized with isoflurane and decapitated. The hemispheres were excised and immediately placed into an ice-cold isolation buffer containing either 125 mM KCl and 10 mM KH_2_PO_4_, pH 7.4 (phosphate medium) or 220 mM mannitol, 70 mM sucrose, 10 mM hepes, 1 mM EGTA, and 0.5% bovine serum albumin (sucrose medium). The cerebellum was removed, and the rest of the brain tissue was cut into small pieces and placed in a 7 mL homogenizer (Duran, Wheaton) with 4 mL of the buffer and homogenized manually for 1 min with 20 strokes of tight-fitting pestle. The brain homogenate was centrifuged at 4000× *g* for 10 min at 4 °C. The pellet was discarded and the supernatant was centrifuged at 12,000× *g* for 15 min at 4 °C. The resulting pellet was resuspended in 0.5 mL of the isolation medium and stored on ice during the experiment. Below, we apply the absolute mice (M) numeration where sucrose medium was used for M1-4 mice and phosphate medium for M5-6 mice.

### 2.2. Mitochondria Functional Assay

The mitochondrial functional assay was carried out in the phosphate medium at room temperature (23 °C). A 20 μM fluorescent dye TMRM (Tetramethylrhodamine, Methyl Ester, Perchlorate, ThermoFisher, Waltham, MA, USA) was used as the mitochondrial membrane potential indicator (the emission peak at 582 nm); 2 mM pyruvate (Sigma Aldrich, St. Louis, MO, USA) was used as a respiration substrate, 100 μM ADP (Sigma Aldrich, USA) was used to activate ATP production, 4 μM mitochondrial oxidative phosphorylation uncoupler CCCP (2-[2-(3-Chlorophenyl) hydrazinylyidene] propanedinitrile, ThermoFisher, USA) was used to render mitochondrial inner membrane permeable to protons.

### 2.3. DT Design

The design principle of the diamond thermometer device was exhaustively described in [1]. Its backbone consists of a glass microcapillary with a low-strain single diamond crystal fixed at the tip of the capillary. The thermosensitivity of DT is provided by an ensemble of silicon-vacancy centers (SiV-centers) embedded in the diamond crystal during the CVD-synthesis. The position of the maximum of the SiV-fluorescence zero-phonon line (ZPL) is sensitive to temperature and provides the ability to record the temperature of any microsystem once calibrated.

### 2.4. DT Calibration

The calibration of the developed DT was performed prior to the experiments with mitochondria. The DT was placed in an air environment for a preliminary determination of the power density of optical excitation at a wavelength that did not lead to a shift of the zero-phonon SiV luminescence line, and, consequently, to heating. The measured power density was used to determine the temperature dependence of the spectral position of the zero-phonon line center in a Linkam TS1500 thermostat stabilized at a predetermined level with an accuracy of 1 °C. The temperature in the chamber was changed in increments of 10 °C. At each step, the SiV luminescence spectrum was recorded and the spectral position of the zero-phonon line maximum was determined according to the algorithm proposed in our previous work [1].

### 2.5. Experimental Setup

The study of the mitochondrial uncoupling thermal response was performed using a LabRam HR800 (Horiba) confocal spectrometer. The SiV fluorescence was excited with a 473 nm laser light (Laser Quantum) which was focused by the low-NA objective to one of the ends of the multimode optical fiber (Thorlabs) with transmission maximum at 740 nm. Another fiber end laid through the interior of the capillary and located near its tip guided the excitation photons directly to microdiamond. The excitation power of the fiber input was chosen to be 3 mW to provide a sufficient fluorescent signal with no additional heating (see Supplementary Materials, Figure S1). The fluorescence was collected with a long-focal-length air objective (Olympus x50, NA = 0.55) and directed to the spectrometer. Probing the mitochondrial viability with TMRM dye was implemented with the same optics equipped with a 532 nm laser and bandpass filter (550–700 nm) in the registration path.

## 3. Results

**Figure 1:**
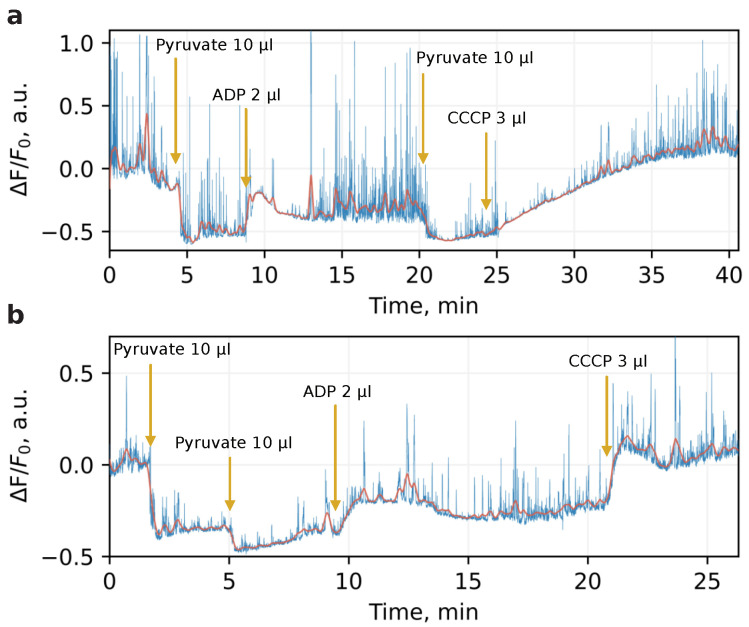
Changes in fluorescence 130 nM TMRM loaded with mitochondria isolated in sucrose (**a**) and phosphate (**b**) medium. A mixture of mitochondria suspension of 150 μL and 1 μL of TMRM was exposed to pyruvate (10 μL), ADP (2 μL), and CCCP (3 μL) action. The difference between the membrane potential reaction to the respiration activation by substrate, its decrease during ATP production, and the loss due to induced proton permeability by CCCP are clearly visible (note that the dye fluorescence decreases with the increase in the potential). The dye fluorescence was excited with a 532 nm laser and collected with the same optics as the SiV fluorescence.

The mitochondria used for studying the local heat production were isolated from six adult mice according to the protocol described in Methods. To verify the viability of the organelles, we examined their bioenergetic state with membrane potential-sensitive dye. For this, immediately after isolation, mitochondria were loaded with TMRM dye which possesses bright and broadband fluorescence with maximum at 580 nm. The time-track of the dye intensity under application of pyruvate, ADP, and CCCP was then recorded. The mitochondrial oxidative phosphorylation uncoupler CCCP renders mitochondrial inner membrane permeable to protons and thus spill off transmembrane potential with transformation of its energy directly to heat. Figure 1 illustrates the typical bioenergetic profile for mitochondria isolated in sucrose (a) and phosphate (b) medium. At specified timepoints, the organelles underwent substrate, ADP, and CCCP application. The fluorescent changes ΔF/F0 were determined as normalized deviations from the baseline before any additions, while the mitochondria isolated in both media demonstrated relevant physiological response reflected by changing of dye intensity with respect to membrane potential. The value of this response was higher in the sucrose medium, more favorable to mitochondrial physiological conditioning. These measurements were performed once for each mouse to verify that mitochondria survived after isolation and displayed a conventional metabolic response.

**Figure 2:**
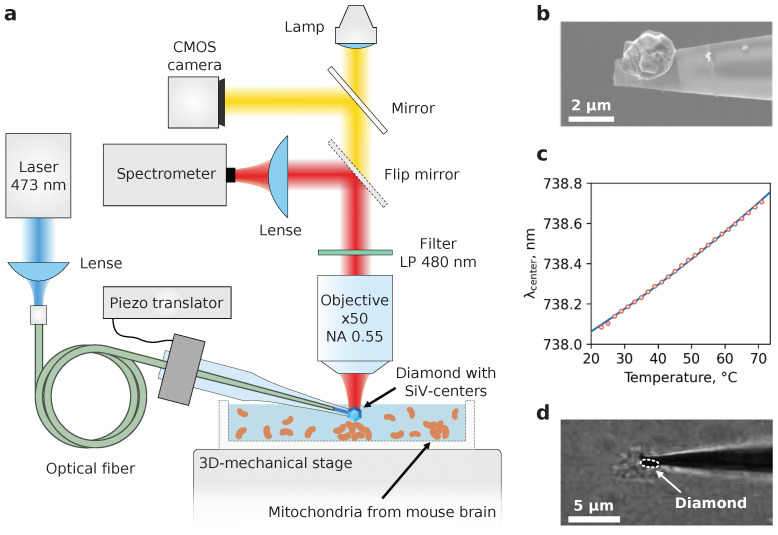
(**a**) Schematic drawing of the experimental setup employed for monitoring the SiV fluorescence and diffusion of mitochondria aggregates. The dish with mitochondrial suspension was coupled to the 3D mechanical stage enabling suitable isolated aggregates of mitochondria to be easily found and visualized by a CMOS optical camera. Independently, the positioning of DT in the focal spot of the objective was governed with a three-axis micromanipulator; (**b**) SEM image of the microdiamond placed at the capillary tip before melting; (**c**) calibration dependence of the ZPL maximum position versus temperature in thermostat; (**d**) representative optical image of the DT stuck to the pre-selected mitochondria aggregate. The white dashed line frames the diamond particle.

Once the viability of the organelles was confirmed, an examination of the mitochondria thermal response to the CCCP electro-chemical action began. A total of 200 μL of mitochondrial suspension was applied to the cap of the Petri dish and smeared on its surface. The rest of the suspension was stored on ice throughout the experiment to preserve mitochondrial metabolism. The dish was then placed on the 3D mechanical stage beneath the microscope objective, and an appropriate aggregate of mitochondria with a size ranging from 2 to 10 μm was found through the optical CMOS camera (see Figure 2d; note that not all visible aggregates are mitochondria since some cell debris still remains after isolation). At the next step the DT was immersed in the solution, and the tip with microdiamond accurately touched the top surface of the targeted aggregate, slightly pressing it. Slight pressure is essential to leave the organelles immobilized during temperature protocols. To induce mitochondrial inner membrane permeability to protons and thus transmembrane potential spill off, 2.5 μL of CCCP was applied to the suspension, and the temperature change was determined. As a reference indication of the macroscopic temperature we used a conventional thermocouple immersed in the solution. Before starting DT recordings, the temperature in the dish was determined as the steady-state ambient temperature (see Supplementary Materials, Figure S2).

**Figure 3:**
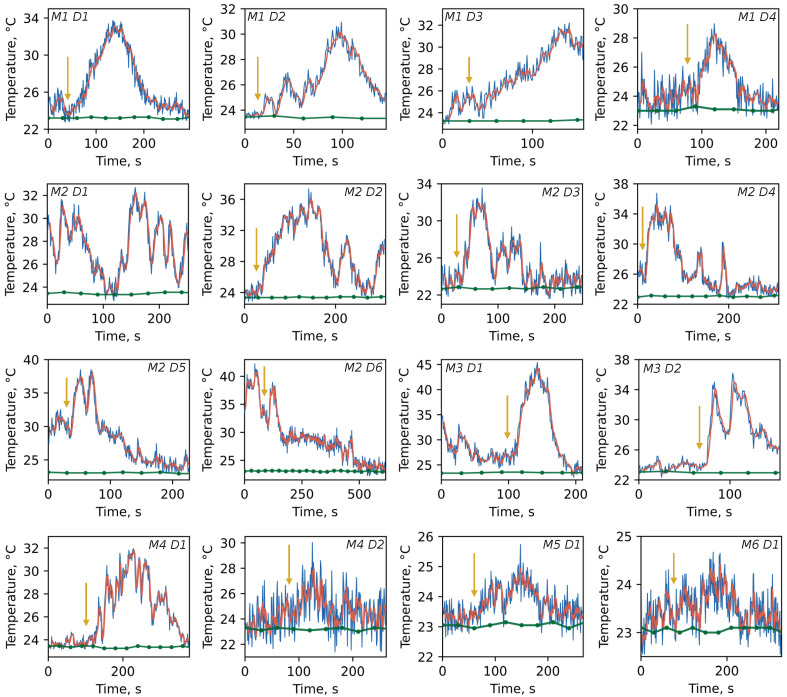
Time tracks of the temperature recorded when DT was in close proximity to individual mitochondria aggregates. The primary data directly measured by DT are indicated in blue and smoothed data are given in red for visual purposes. The green curves are thermocouple temperature readings of the whole solution. The yellow arrows specify the moment of CCCP injection (2.5 μL). The text at the right or left-top corners specifies the mice (M) and dish (D) number for which the temperature was measured. Note that the dish numbers are shown in chronological sequence with an average time between each one being approximately 20 min. The difference in the noise level is related to different thickness of the suspension layer and, therefore, dissimilar turbidity of the suspension.

Figure 3 illustrates the temperature tracks recorded for different dishes within each of the six mice. The time step between DT indications was chosen to be 1 s to achieve the best signal-to-noise ratio while still considering the possible quick thermal dynamics. One can see that the temperature elevation ΔT ranges from several to tens degrees Celsius above the ambient level (green dots) primarily right after CCCP injection (yellow arrow). The maximum thermal burst within a single dish of $\Delta T_{max}=22.4$ °C is observed for M3D1. However, temperature bursts were also detected before CCCP application (e.g., M2D1, M2D6, M3D1). Such spontaneous thermal responses are not lower in amplitude than CCCP-stimulated ones and could be attributed to the mitochondria which may be currently involved in ATP synthesis or controlled potential leaking to avoid reactive oxygen species hyperproduction due to excessive potential value.

**Figure 4:**
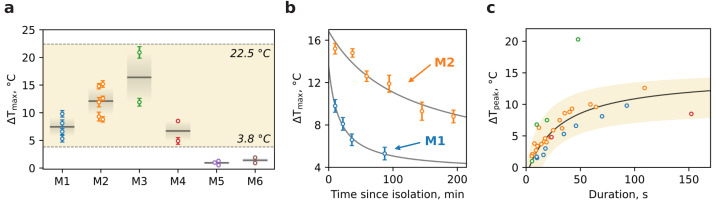
Statistics and kinetics of thermal responses per mice. (**a**) Maximal thermal response $\Delta T_{max}$ for a variety of mice. The gray lines indicate the mean value over dishes for selected mice; the same color gradients extend the standard deviation. The vertical dashed line separates two groups of mitochondria: M1–M4 in the glucose-containing medium and M5–M6 in the glucose-free one. (**b**) Time evolution of $\Delta T_{max}$ for mice M1 and M2. Gray solid lines are added for visual purposes. (**c**) The amplitude of thermal bursts $\Delta T_{peak}$ collected from temperature traces as a function of their duration. Note that $\Delta T_{peak}$ takes into account the background baseline humpback. Experimental points were fitted with black curve.

The burst duration also varies in a wide range from seconds to hundreds of seconds (Figure 4b), probably due to inhomogeneous distribution of CCCP action on individual mitochondria within one aggregate: while mitochondria on the edge of aggregate are exposed to the uncoupler action, the deep-dwelling organelles are isolated and remain unperturbed. Thus, the slow penetration of CCCP leads to an asynchronous and heterogeneous time response.

An analysis of the temperature traces allowed us to obtain the $\Delta T_{max}$ values per dish for a variety of mice (Figure 4a). The mean thermal response of mitochondria for M1–M4 lies at the level of $<\Delta T_{max}>\;∼$11 °C though individual traces show an outstanding temperature breakthrough of 15–22 °C. In contrast, traces for M5 and M6 demonstrate smaller amplitude dynamics with mean $<\Delta T_{max}>\;∼$1 °C. The possible explanation of this behavior is based on the difference between mitochondria isolation media. The sucrose- and mannitol-containing medium with EGTA and BSA is more favorable to mitochondrial physiological conditioning during the isolation period due to avoidance of excessive K^+^ and phosphate load, Ca^2+^ ions chelation, and free lipids absorbance, which altogether leads to a higher yield of intact mitochondria with high metabolic capabilities.

Figure 4b demonstrates the alteration of $\Delta T_{max}$ in time for M1 and M2 with an apparent decrease for both which is obviously due to mitochondria degradation as the time passed. However, the attenuation rate for M1 is slightly faster than that for M2. So, at 90 min thermal response the amplitude reduces two times for M1 and only 1.3 times for M2. This dissimilarity may be determined by many factors including age and physiological condition of mice or slight differences during isolation, especially at the homogenization phase.

Ultimately, we delve into the kinetics of thermal responses for M1–M4 mice (Figure 4c). Each burst in temperature traces presented in Figure 3, either spontaneous or CCCP-stimulated, was carefully treated in order to evaluate temperature amplitude $\Delta T_{peak}$ in accordance with its duration determined as full-width at half-maximum (FWHM). The set of points calculated for each mouse is shown in Figure 4c. The dependence clearly illustrates the saturation nature of thermal bursts and therefore was fitted (black curve) with $\Delta T_{peak}\left(\tau \right)=\Delta T_{peak}^{\infty }\cdot \tau /\left(\tau +\tau_{sat}\right)$, where $\Delta T_{peak}$ is a temperature long-term limit, $\tau_{sat}$ is the saturation duration. An approximation gives $\Delta T_{peak}=14.5$ °C and $\tau_{sat}=33$ s implying the principal amplitude of temperature bursts ceases to increase significantly at the duration of ~30 s. Apparently, this behavior implies that the resource of potential energy accumulated in mitochondria is limited and not enough to produce heat permanently.

## 4. Discussion

### 4.1. CCCP-Induced Heat Production

Our experiments show that the transmembrane potential spills out under CCCP application leads to a heating of the mitochondria by 4–22 °C above the ambient temperature.

Such a broad variation in the temperature response may be associated with the heterogeneity of the mitochondria themselves as well as their aggregations in the isolated suspension. It is in accordance with the literature data on different mitochondrial types in neurons [22]. It was shown that small axonal mitochondria provide Ca ions bufferization to maintain synapse transduction [23]. Dendrite mitochondria are rather elongated, and the soma mitochondria form a kind of an organelle network [24]. There are data that suggest that mitochondria isolated from neuronal and glial cells differ by several physiological parameters [25]. Mitochondria from cells with greater energy demand have more cristae and less mitochondrial matrix volume. Therefore, the thermal profiles of mitochondria from different cellular and subcellular locations can be fundamentally different. Furthermore, the spatial distance between the DT active element and organelle aggregates changed randomly that could contribute to observed constant temperature dissimilarity, as the temperature gradient around them was rather steep.

The maximum absolute temperature that was recorded in our experiments was 45 °C. This value is close to 50 °C reported in the revolutionary work of Chre’tien et al. [15] and is the first independent confirmation of these results. In their work, two cell types were used: those of the human embryonic kidney (HEK) 293 line and primary skin fibroblasts. For the first time, we report such high temperature values for brain mitochondria. Taken together, ours and Chre´tien’s data suggest universal mitochondrial characteristics throughout all tissue types.

Rather intriguing is the temperature rise ceiling being apparently independent from the ambient temperature and cell type. In Chre´tien et al. [15], it was 50 °C vs. 38 °C, and in our work, it is 45 °C vs. 23 °C. In these cases, the rise amplitude is different: is it due to various energy accumulations in the mitochondria in different experiments or rather due to a limited temperature endurance of the mitochondrial molecular machinery related to some kind of regulatory mechanisms keeping it in physiological boundaries? Does it yet vary depending on cell types or the subcellular location? Further experiments are required to answer these questions.

The burst duration varies in a wide range from seconds to hundreds of seconds (Figure 4b). This may be due to the well-known heterogeneity of individual mitochondria in physical size, functional state, energy capacity, and other physiological parameters. Another source of the variation could be related to inhomogeneous CCCP action on individual mitochondria within aggregates: while mitochondria on the edge of an aggregate are exposed to the uncoupler action, the deep-dwelling organelles are isolated and remain unperturbed. Thus, the slow penetration of CCCP leads to asynchronous and heterogeneous time response.

Taken together, this could explain the amplitude of thermal bursts as a function of their duration (Figure 4c). In the low range below 50 s it shows a nearly linear dependency with k 0.3 °C/s and may be due to individual mitochondria variations, whereas at longer times, the aggregate dynamics could come into play, lengthening the response duration while the absolute temperature limit is already reached. The apparent outlier in M3 is probably an analysis artifact due to the responses blended together and treated as a single one, although an exceptionally potent individual mitochondrion cannot be ruled out.

### 4.2. Physiological Heat Production

Mitochondria are powerful and specialized factories for transforming the energy of the aerobic respiration process to the universal cellular energy equivalent in the form of macroergic phosphate bonds of ATP. Possessing a rather low coefficient of energy conversion efficiency of 40–60%, they are natural producers of heat. They also maintain a decoupling mechanism that is used to control the upper value of the proton potential in order to avoid excessive production of reactive oxygen species. This mechanism renders the membrane permeable to protons and drops the potential with the releasing of heat due to the direct transformation of the electrochemical gradient energy to the thermal energy and serves as a preadaptation to the development of homeothermy that maintains organism homeostasis and intensifies metabolism to an unprecedented level. Thus, in normal physiological conditions we expect an increase in mitochondrial thermogenesis during ATP production and also in mitochondria with a good respiration substrates supply under the lack of ADP stock.

This is presented in our experiments as temperature bursts that were detected prior to CCCP application (e.g., M2D1, M2D6, and M3D1). Such spontaneous thermal responses could be attributed to the mitochondria which may be currently involved in ATP synthesis or controlled potential leaking to avoid the hyperproduction of reactive oxygen species due to excessive potential value.

## 5. Conclusions

Production of heat by mitochondria is critical for maintaining body temperature, regulating metabolic rate, and preventing oxidative damage to mitochondria and cells. Until now, mitochondrial heat production was measured only by fluorescent probes which are sensitive to environmental variations. Here, the temperature of isolated mitochondria was unambiguously measured by a diamond thermometer that is absolutely indifferent to external non-thermal parameters. Under uncoupling of transmembrane potential by CCCP application, the temperature near the mitochondria rises by 4–22 °C above the ambient with an absolute maximum of 45 °C. Such a broad variation in the temperature response is associated with the heterogeneity of the mitochondria themselves as well as their aggregations in the isolated suspension. Spontaneous temperature bursts with the comparable amplitude were also detected prior to CCCP application which can reflect involvement of some mitochondria to ATP synthesis or membrane potential leaking to avoid hyperproduction of reactive oxygen species.

The proposed diamond temperature sensor as a direct measurement instrument and the data obtained can greatly advance studies of thermodynamics at the subcellular level. In addition to fundamental applications, the new methodological approach may finally reveal the unclear mechanisms underlying temperature dysregulation in normal conditions and pathologies such as those of the central nervous system, which directly depend on the functional state of mitochondria.

## Data Availability

Not applicable.

## Supplementary Materials

The following supporting information can be downloaded at: https://www.mdpi.com/article/10.3390/nano13010098/s1. Figure S1. A power dependence of the temperature detected by DT in the dish without mitochondria. The indicated power is measured before fiber input; Figure S2. A control time-track of the temperature in the dish without mitochondria.
