# Supplementary material for: Heat Release by Isolated Mouse Brain Mitochondria Detected with Diamond Thermometer
Source: Nanomaterials (Basel). 2022 Dec 25;13(1):98. doi: 10.3390/nano13010098 (PMC9823591; doi:10.3390/nano13010098)
Supplement: Supplementary file 1 [file nanomaterials-13-00098-s001.zip › nanomaterials-2087354-supplementary.pdf]

Heat release by isolated mouse brain mitochondria detected with diamond thermometer

Supplementary information

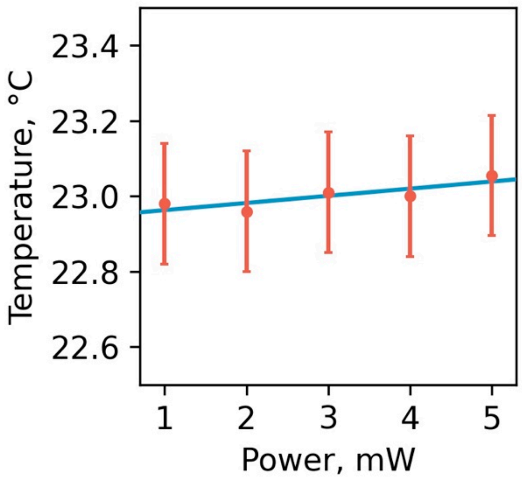

Figure S1. A power dependence of the temperature detected by DT in the dish without mitochondria. The indicated power is measured before fiber input.

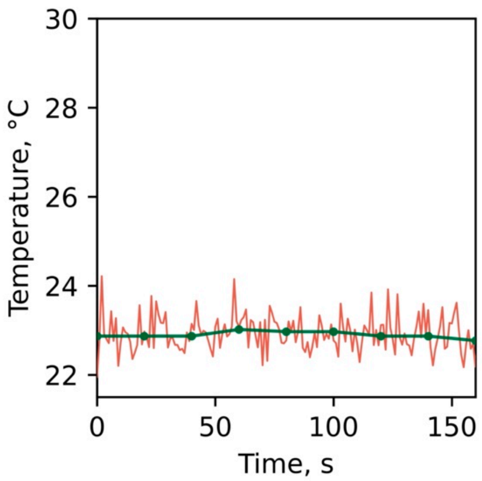

Figure S2. A control time-track of the temperature in the dish without mitochondria.
